# New therapeutic approaches for Krabbe disease: The potential of pharmacological chaperones

**DOI:** 10.1002/jnr.23762

**Published:** 2016-09-17

**Authors:** Samantha J. Spratley, Janet E. Deane

**Affiliations:** ^1^ Cambridge Institute for Medical ResearchDepartment of Pathology University of CambridgeCambridgeUnited Kingdom

**Keywords:** Krabbe disease, pharmacological chaperone therapy, β‐galactocerebrosidase, GALC, lysosomal storage disorder, galactocerebroside

## Abstract

Missense mutations in the lysosomal hydrolase β‐galactocerebrosidase (GALC) account for at least 40% of known cases of Krabbe disease (KD). Most of these missense mutations are predicted to disrupt the fold of the enzyme, preventing GALC in sufficient amounts from reaching its site of action in the lysosome. The predominant central nervous system (CNS) pathology and the absence of accumulated primary substrate within the lysosome mean that strategies used to treat other lysosomal storage disorders (LSDs) are insufficient in KD, highlighting the still unmet clinical requirement for successful KD therapeutics. Pharmacological chaperone therapy (PCT) is one strategy being explored to overcome defects in GALC caused by missense mutations. In recent studies, several small‐molecule inhibitors have been identified as promising chaperone candidates for GALC. This Review discusses new insights gained from these studies and highlights the importance of characterizing both the chaperone interaction and the underlying mutation to define properly a responsive population and to improve the translation of existing lead molecules into successful KD therapeutics. We also highlight the importance of using multiple complementary methods to monitor PCT effectiveness. Finally, we explore the exciting potential of using combination therapy to ameliorate disease through the use of PCT with existing therapies or with more generalized therapeutics, such as proteasomal inhibition, that have been shown to have synergistic effects in other LSDs. This, alongside advances in CNS delivery of recombinant enzyme and targeted rational drug design, provides a promising outlook for the development of KD therapeutics. © 2016 The Authors. Journal of Neuroscience Research Published by Wiley Periodicals, Inc.

Krabbe disease (KD; also known as *globoid cell leukodystrophy*) is an autosomal recessive sphingolipidosis with a severe and progressive neurodegenerative disease course caused by deficiencies in the lysosomal hydrolase β‐galactocerebrosidase (GALC). GALC is required for the hydrolysis of galactosphingolipids, including the major lipid component of myelin β‐galactocerebroside (GalCer) required for lipid turnover and maintenance of the myelin sheath that surrounds and protects neurons. Although KD is classified as a lysosomal storage disorder (LSD), unlike other LSDs, there is little to no storage of primary substrate (Eto and Suzuki, 1970). Instead, GALC deficiency results in the progressive accumulation of the cytotoxic galactosphingolipid substrate psychosine (also known as *galactosylphingosine*; Svennerholm et al., 1980; Igisu and Suzuki, 1984). UDP‐galactose:ceramide galatosyltransferase 8 catalyzes the synthesis of both GalCer and psychosine through the galactosylation of ceramide and sphingosine, respectively (Suzuki, 2003). Under normal physiological conditions, psychosine levels in the brain are virtually undetected. However, in KD, GALC is unable to break down the cytotoxic psychosine, and its accumulation is widely accepted to be responsible for the cell death associated with the rapid and widespread demyelination throughout both the CNS and the peripheral nervous system observed in KD (Suzuki, 1998; Won et al., 2013). Psychosine accumulation has been reported to perturb lipid raft architecture, impede oligodendrocyte differentiation, and cause aberrant cell signaling; however, the precise mechanisms of cell death remain unclear (Giri et al., 2006; Ballabio and Gieselmann, 2009; White et al., 2009; Won et al., 2013).

Newly synthesized GALC is cotranslationally translocated into the endoplasmic reticulum (ER) and enters the Golgi apparatus, where it is posttranslationally modified by N‐linked glycosylation at four sites (Deane et al., 2011; Fig. 1). Glycan side chains are further modified by the addition of mannose 6‐phosphate (M6P) groups that are recognized by the cation‐independent M6P receptor (M6PR). The M6PR–GALC complex is transported from the trans‐Golgi network (TGN) to the endosomal compartments. In the low‐pH environment of the late endosome, the M6PR–GALC complex dissociates, the M6PR is recycled back to the TGN, and GALC is delivered to the lysosome (Gu et al., 2001; Huotari and Helenius, 2011). Alternatively, GALC can be delivered to the lysosome indirectly via secretion and reuptake by the M6PR (Nagano et al., 1998). Only proteins that are correctly folded and stable can exit the quality control systems within the ER and traffic correctly to the lysosome. After successful delivery to the lysosome, a loop on the surface of GALC is cleaved, resulting in the detection of 50‐kDa and 30‐kDa bands by SDS‐PAGE. However, this cleavage event is not an activating step because the uncleaved enzyme retains activity (Hill et al., 2013). GALC has optimal catalytic activity at low pH and processes sphingolipid substrates in the acidic environment of the lysosome. Nonenzymatic sphingolipid activator proteins known as *saposins* are additionally required for the degradation of sphingolipids by lysosomal hydrolases. In rare cases, lack of functional saposin A can cause KD (Spiegel et al., 2005; Table 1).

**Figure 1:**
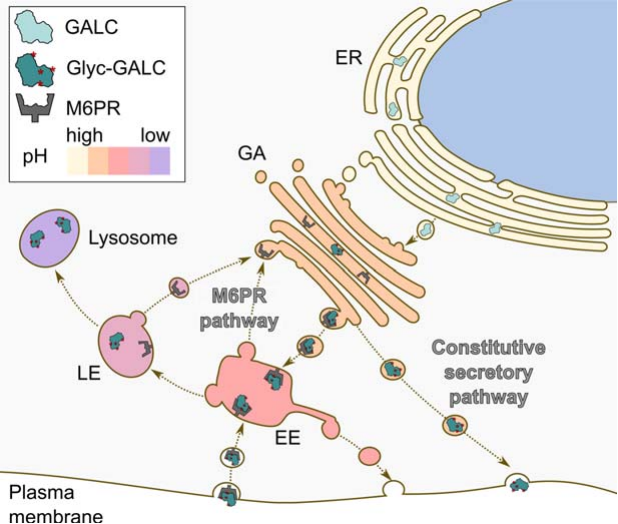
Processing and trafficking of GALC to the lysosome. GALC is produced in the ER and glycosylated (Glyc‐GALC) in the Golgi apparatus (GA). Glycans modified with M6P groups are recognized by M6PR. The M6PR‐GALC complex is transported to the early endosomal (EE) compartment, and, in the low‐pH environment of the late endosome (LE), the complex dissociates. The M6PR is recycled back to the GA, and GALC is delivered to the lysosome. GALC can also be trafficked via the constitutive secretory pathway and delivered to the lysosome via reuptake by the M6PR.

**Table 1:** Glossary and Abbreviations

Abbreviation	Commonly used names	Description
Allosteric chaperone	Non‐active site chaperone	A small molecule that modulates enzyme activity by binding to a site other than the active site
α‐Gal A	α‐Galactosidase A	Lysosomal glycoside hydrolase, responsible for the hydrolysis of terminal α‐galactosyl moieties from glycolipids including globotriaosylceramide; deficient in Fabry's disease
Azasugar	Iminosugar	A sugar derivative possessing a nitrogen atom in the ring of the structure
β‐Gal	β‐Galactosidase	Lysosomal glycoside hydrolase, responsible for the hydrolysis of β‐galactosides; deficient in GM_1_ gangliosidosis and Morquio B disease
BBB	Blood–brain barrier	A highly selective, permeable membrane that provides a dynamic interface between the brain and the circulatory system to protect the CNS
DGJ	1‐Deoxy‐*galacto*‐nojirimycin; migalastat	Nonalkylated azasugar; reversible competitive inhibitor of α‐galactosidase A
DNJ	1‐Deoxynojirimycin	Glucose configured azasugar
ERAD	Endoplasmic reticulum associated degradation	A cellular pathway that targets misfolded proteins in the ER for subsequent degradation by the proteasome
ERT	Enzyme replacement therapy	Therapeutic administration of an enzyme that is defective or missing in a patient to alleviate the effects of enzyme deficiency
GALC	β‐Galactocerebrosidase; galactosylceramidase	Lysosomal glycoside hydrolase, responsible for the hydrolysis of terminal β‐galactosyl moieties from glycolipids, including galactosylceramide, psychosine, and lactosylceramide; deficient in KD
GalCer	Galactocerebroside; galactosylceramide	GALC natural substrate, a cerebroside consisting of a ceramide with a galactose residue
HMG	6‐Hexadecanoylamino‐4‐methylumbelliferyl‐β‐D‐galactopyranoside	Alkylated fluorogenic GALC substrate
HNG	2‐Hexadecanoylamino‐4‐nitrophenyl‐β‐D‐galactopyranoside	Alkylated chromogenic GALC substrate
IGF	Iso‐*galacto*‐fagomine; 4‐epi‐isofagomine	Nonalkylated azasugar; galactosidase inhibitor
KD	Krabbe disease; globoid cell leukodystrophy	Lysosomal storage disorder caused by deficient β‐galactocerebrosidase; characterized by a progressive neurodegenerative disease course
LSD	Lysosomal storage disorder	A family of rare inherited disorders caused by gene mutations that disrupt lysosomal function; typically characterized by abnormal accumulation of substrate within the lysosome
NB‐DNJ	N‐butyl deoxynojirimycin; miglustat; zaveska	Alkylated glucose configured azasugar; ceramide glucosyltransferase and GAA I and II inhibitor
PCT	Pharmacological chaperone therapy	A therapeutic strategy that uses small‐molecule inhibitors competitively and reversibly to bind and stabilize the native conformation of misfolded proteins
SRT	Substrate reduction therapy	Use of inhibitors to reduce the synthesis of substrates such that residual degradative activity is sufficient to prevent substrate accumulation
4MβDG	4‐Methylumbelliferyl‐β‐D‐galactopyranoside	Water‐soluble fluorogenic GALC substrate
4NβDG	4‐Nitrophenyl‐β‐D‐galactopyranoside	Water‐soluble chromogenic GALC substrate

## THERAPEUTIC STRATEGIES FOR KD

Just as with many other LSDs, the molecular heterogeneity and poor correlation among genotype, phenotype, and prevalence of CNS involvement pose a significant barrier for successful therapeutic intervention for KD. Currently, the only approved and available treatment option for those affected with KD is hematopoietic stem cell transplantation (HSCT; Krivit et al., 1998). However, its limited success, short therapeutic window, and high associated risk highlight the still unmet clinical requirement for other viable treatment options (Siddiqi et al., 2006; Duffner et al., 2009).

Enzyme replacement therapy (ERT) is an approach commonly used to treat LSDs and is the current standard of care for Gaucher's, Fabry's, and Pompe's diseases as well as for mucopolysaccharidoses (MPS) I, II, and VI (Sands, 2014). However, the primary neurodegeneration observed in KD requires the desired therapeutic to cross the blood–brain barrier (BBB), and recombinant lysosomal enzymes used for ERT are too large to achieve this. The transient nature of ERT also requires weekly administrations of recombinant enzyme, which are expensive in both time and resources. The recent advances made in gene therapy provide a promising avenue for the treatment of CNS‐involved LSDs. Viral vectors such as AAV9 have been shown to cross the BBB for effective CNS delivery in MPS III A and B (Foust et al., 2009; Fu et al., 2011; Aronovich and Hackett, 2015). For KD, it has been suggested that specific neuronal or glial tropisms may be required in such viral vectors, and work with the twitcher mouse model of KD is showing great promise (Rafi et al., 2012). This is an exciting direction for new therapeutic approaches for KD but is outside the scope of this Review and has been well reviewed elsewhere (Hawkins‐Salsbury et al., 2011; Nagabhushan Kalburgi et al., 2013).

Substrate reduction therapy (SRT) is another approach that has shown promise in treating LSDs such as in type I Gaucher's disease (Cox et al., 2000, 2003). By decreasing the synthesis of primary substrate, SRT seeks to target and diminish pathogenic substrate accumulation and reduce lysosomal dysfunction as a result of decreased load. However, because accumulation of the primary substrate GalCer is not observed in KD, the benefits of reducing substrate production in KD remain unclear.

## CHAPERONE THERAPY FOR KD

**Figure 2:**
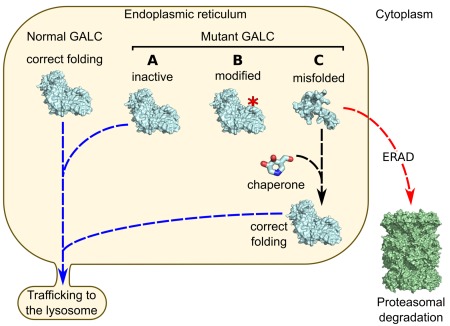
Schematic representation of mutant protein fate within the cell and the effect of small‐molecule chaperones. In a healthy individual, GALC is synthesized and correctly folded within the ER and subsequently transported to its site of action in the lysosome. Missense mutations in GALC can cause an array of deleterious effects that lead to KD. Missense mutations can affect the active site of the enzyme and lead to catalytic deficiencies (A), cause posttranslational modifications that can mistarget GALC within the cell (B), or destabilize and misfold GALC (C). Misfolded GALC is retained in the ER, where it is targeted by ERAD and degraded, leading to loss of functional GALC reaching the lysosome. Small‐molecule chaperones can bind to and stabilize GALC to overcome incorrect folding and escape degradation, allowing successful transport to the lysosome.

GALC missense mutations are responsible for at least 40% of known KD cases, and most of these are predicted to disrupt the fold of the enzyme (Deane et al., 2011). Mutations that result in improper enzyme folding mean that these mutant enzymes are not able to escape the tight quality control systems within the ER and are consequently retained in the ER, where they are targeted for degradation by ER‐associated degradation (ERAD) or, in some cases, incorrectly modified and subsequently mistargeted within the cell (Fan, 2003). All such fates lead to a loss of functional enzyme within the lysosome and can elicit pathogenesis. In these cases, an alternative treatment option currently being explored is pharmacological chaperone therapy (PCT). PCT relies on the use of small molecules that specifically bind to and favor the stabilization of the native conformation of misfolded proteins, with the view of overcoming ER retention and associated degradation and facilitating delivery of enzymes to their required site of action (Ulloa‐Aguirre et al., 2004; Fig. 2). The ideal chaperone would bind to a misfolded enzyme while it is synthesized in the ER, stabilize the enzyme's native conformation, and chaperone the enzyme to its site of action in the lysosome. Here, the chaperone would be displaced either by excess of natural substrate or by pH effects, allowing the enzyme to carry out its normal function. An advantage of using a chaperone approach for lysosomal protein dysfunction is the potential of harnessing the acidic environment of the lysosome for chaperone dissociation, whereby chaperone–enzyme complexes can be stably transported to the lysosome and then dissociate in the acidic conditions (Suzuki, 2013). By assisting in the folding and stability of mutant enzymes, the aim of PCT is to overcome lysosomal enzyme deficiency by increasing the pool of functional enzymes reaching the desired site of action (Parenti, 2009).

The effectiveness of pharmacological chaperones (PCs) is typically monitored by an increase in specific hydrolase activity. Partial restoration of enzyme activity to only 10% of wild‐type (WT) levels has been reported to provide sufficient enzyme activity to prevent disease in LSDs (Kolter and Sandhoff, 1998). Therefore, even a small increase in the amount of functional enzyme reaching the lysosome could significantly attenuate disease progression (Leinekugel et al., 1992; Kolter and Sandhoff, 1998). Several Krabbe‐associated missense mutations, such as Y551S, G270D, and [I66M + I289V] (Xu et al., 2006; Tappino et al., 2010), have been reported to retain residual enzyme activity. This, together with the low level of enzymatic activity required to prevent disease in other lysosomal disorders, identifies PCT as a promising approach for KD (Schueler et al., 2004).

Penetration of the BBB is a major limitation for the development of LSD therapy because 75% of described LSDs have significant CNS involvement (Sands, 2014). The properties of some small‐molecule chaperones (low molecular weight, low toxicity, and high bioavailability) lend themselves well for the treatment of CNS pathology because they have a greater capacity to cross the BBB than enzymes. However, to achieve pharmacologically significant CNS delivery, additional properties, such as high lipid solubility, are also required (Pardridge, 2005). In other LSDs, low‐molecular‐weight competitive inhibitors that function as PCs to restore catalytic activity of mutant enzymes have been identified (Suzuki et al., 2009). Several of these candidates have led to successful clinical translation, including but not limited to migalastat (Amicus, Cranbury, NJ) for Fabry's disease (Germain et al., 2012), ambroxol (ExSAR, Monmouth, NJ) for type I Gaucher's disease (Zimran et al., 2013), and pyrimethamine for GM_2_ gangliosidosis (Clarke et al., 2011). Although these have provided proof of principle for PCT as a viable treatment option for LSDs, the limited clinical translation of PCT to date means that their utility as a therapeutic is unclear, and, consequently, their long‐term efficacy remains uncertain. Many of these have been very well reviewed elsewhere (Valenzano et al., 2011; Boyd et al., 2013; Shayman and Larsen, 2014; Parenti et al., 2015a, 2015b), so this Review will focus on the development of PCT specifically for KD.

## IDENTIFYING CANDIDATE CHAPERONES FOR KD

Multiple approaches have been explored to identify molecules that exert a chaperoning effect on misfolded proteins in LSDs. The most common approaches involve the use of substrate mimics that are often targeted against the active site of the deficient hydrolase, permitting specificity and limiting off‐target deleterious effects. Alternatively, allosteric chaperones have been identified that are principally targeted away from the active site of the enzyme. As such, this class of chaperone circumvents the problems associated with enzyme inhibition often encountered with substrate mimics. In a more general approach, cellular pathways often associated with misfolded proteins such as ERAD and proteostasis have been targeted to promote general protein folding. This latter approach has the potential to target the secondary changes that often contribute to the complex nature of LSDs and may, in part, address the mutation‐dependent effects often observed with chaperones in heterogeneous genetic disorders. Several of these approaches have been used for the identification of GALC candidate chaperones for KD and are reviewed here.

### Substrate Mimics

Chaperone molecules used for the correction of misfolded proteins in LSDs are often active‐site‐specific competitive inhibitors. Targeting the active site of the deficient hydrolase allows enhanced specificity, reducing the risk of off‐target effects, and can limit inhibitory action on other, related enzymes (Butters et al., 2005). Azasugar derivatives (also referred to as *iminosugars*) are structural analogs of the sugar moieties on sphingolipid substrates; as such, they make up a family of potent glycosidase inhibitors and have been widely explored as candidate chaperones for LSDs (Fan, 2007). As well as being highly soluble, azasugars have excellent biodistribution and low toxicity and have been pursued as a promising class of active‐site‐directed chaperone (Mellor et al., 2004; Treiber et al., 2007; Horne et al., 2011). One of the first to be described, 1‐deoxygalactonojirimycin (DGJ; migalastat), illustrates the potential of azasugars as chaperone candidates for LSDs. DGJ is an inhibitor of α‐galactosidase A (α‐gal A), the defective enzyme in Fabry's disease and has subsequently reached phase III clinical trials (Fan et al., 1999; Guce et al., 2011; Germain et al., 2012; Ishii, 2012).

In a recent study, a series of azasugar‐based galactoside mimics was designed, synthesized, and evaluated for their inhibitory effect on GALC (Biela‐Banas et al., 2014). To assess specificity for GALC, these studies were carried out alongside the related lysosomal enzymes α‐gal A and β‐galactosidase (β‐gal). Total leukocyte sonicate was used as the source of enzyme for these assays, and specificity of inhibition was determined through use of the substrate molecules tritiated GalCer for GALC, 4‐methylumbelliferyl (4MU)‐β‐D‐galactopyranoside (4MBDG) for β‐gal, and 4MU‐α‐D‐galactopyranoside for α‐gal A. Chaperone suitability was determined by the percentage inhibition conferred by each of the tested molecules on GALC at lysosomal pH 4 compared with its effects on α‐gal A and β‐gal.

**Figure 3:**
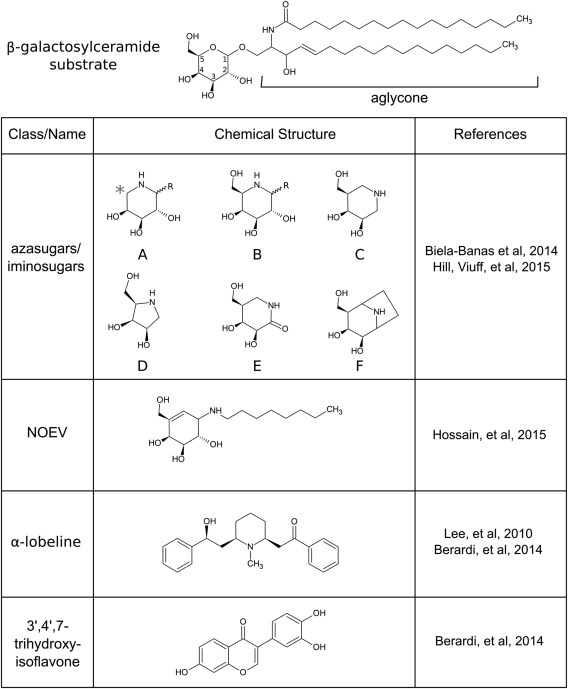
Different classes of PCT molecules identified for KD. **Top**: Chemical structure of the primary GALC substrate β‐galactosylceramide illustrating atom numbering for the glycosyl moiety. **Bottom**: Classifications and chemical structures of small molecules identified as potential PCT candidates for KD. Specific azasugar molecules include 1‐C‐alkyl imino‐L‐arabitols (A); 1‐C‐alkyl imino‐D‐galactitols, e.g., DGJ (B); 1‐N‐iminosugars, e.g., IGF (C); DIL (D); IGL (E); and DGN (F).

This study demonstrated several important features of the azasugar candidates critical to their function as GALC inhibitors. First, by testing 1‐C‐alkyl imino‐L‐arabitols (Fig. 3, A), the 5‐CH_2_OH group (marked by an asterisk) was shown to be an essential constituent for both α‐gal A and β‐gal inhibition, supporting previous observations (Bernotas et al., 1990). Second, it was demonstrated that the 1‐C‐alkyl imino‐D‐galactitols (as with DGJ; Fig. 3, B) were more potent inhibitors of α‐gal A than of the β‐gals, whereas the 1‐N‐iminosugar series (Fig. 3, C) was found to be the most potent inhibitor of the β‐gals. This critically highlights the importance of the position of the nitrogen atom in the azasugar ring for specificity between α‐ and β‐gals. Although 4‐epi‐isofagomine (also known as *iso‐galacto‐fagomine*; IGF) was identified as a GALC inhibitor and conferred the greatest inhibition on the β‐gals, its inhibitory effect on β‐gal identifies a lack of specificity that may compromise its potential as a KD therapeutic. However, Biela‐Banas et al propose that modifications of IGF may result in similar inhibition with increased GALC specificity. Although these findings highlight the subtleties in the azasugar ring architecture that are required for specificity and inhibition, an understanding of each compound's stabilizing effect on GALC at both lysosomal and ER pH (representing the desired cellular conditions of initial chaperone binding) would be beneficial, given that it is widely known that binding can vary greatly between these two cellular environments (Lieberman et al., 2009).

By targeting the active site of the enzyme, chaperones can be rationally designed to enhance specificity. Hill et al. (2015) used insights gained from GALC structural data as an atomic framework to design and characterize a series of azasugar derivatives to serve as GALC‐specific candidate chaperones. Six different azasugars were tested, including the nonalkylated azasugars DGJ and IGF (Fig. 3, B, C); a related hydrazine aza‐*galacto*‐fagomine (AGF) possessing two nitrogen atoms in the sugar ring equivalent to those in both IGF and DGJ; a five‐membered pyrrolidine derivative, dideoxy‐imino‐lyxitol (DIL; Fig. 3, D); a δ–lactam derivative, iso‐*galacto*‐fagomine lactam (IGL; Fig. 3, E); and a bicyclic nortropane, deoxy‐*galacto*‐noeurostegine (DGN; Fig. 3, F). Inhibitory kinetics were determined by using purified GALC for each of these candidates at pH 4.6, the optimal pH for GALC activity. With the exception of the bridged azasugar DGN, all of those tested exhibited significant competitive inhibition of GALC. This study was extended beyond inhibition to illustrate the ability of each of the molecules to confer stabilization of GALC in vitro and to show that the observed stabilization is directly correlated with binding affinity. IGF and AGF were the most potent inhibitors (*K*_i_ 380 ± 26 nM and 630 ± 53 nM, respectively) and conferred the highest degree of global stabilization, providing the basis for using these as optimal scaffolds. GALC stabilization was measured by thermal denaturation at two different pH values representing the cellular environments of the lysosome (pH 4.6) and ER (pH 7.2). All but one test compound (DGN) increased the stability of WT enzyme under both conditions in a dose‐dependent manner. However, stabilization of GALC was greater at low pH than at neutral pH, a suboptimal feature for a PC. To confirm specificity, glucose‐configured 1‐deoxynojirimycin (DNJ) was tested and did not stabilize or change the melting temperature of GALC. Crystallographic studies showed that each of the candidate compounds directly bound in the active site of GALC. Analysis of the binding interactions revealed the critical role of the interaction between the ring nitrogen and the side chain of the active site nucleophile (E258) as well as the importance of the ring pucker. This study reinforces the greater potency of IGF over other azasugar derivatives and highlights the specific characteristics of azasugars required for optimal binding specificity and affinity. However, this study also reveals that, because of the electrostatic nature of the interaction, azasugar molecules bind more tightly at lysosomal pH than they do at neutral pH. Thus, PCT molecules that contain nitrogen in the ring have the advantage of high‐affinity binding but have to overcome the tighter binding at low pH to function as efficient chaperones. In these cases, rather than exploiting the acidic pH of the lysosome, the success of these molecules may depend on the ability of excess substrate to compete for binding to the active site.

Another molecule resembling the natural substrate of GALC is N‐ocytl‐4‐epi‐b‐valienamine (NOEV; Fig. 3), a potent β‐gal inhibitor and potential chaperone therapeutic for GM_1_‐gangliosidosis (Suzuki et al., 2007). Hossain et al. (2015) tested NOEV as a candidate chaperone for mutant GALC, with an emphasis on those mutations attributed to late onset forms of KD. To determine whether NOEV could confer resistance to heat denaturation, enzyme activity was measured in lysates from GALC‐transfected cells that had been incubated at 48 °C in the presence and absence of NOEV. After heat treatment, WT GALC lysates retained higher activity in the presence of NOEV, suggesting that this molecule increases the stability of GALC. However, lysates from six different mutant forms of GALC did not display significant stabilization with NOEV. An alternative measure of successful chaperoning of GALC to the lysosome is to monitor the appearance of the cleaved form of GALC that occurs upon delivery to the lysosome. For several GALC variants, including G270D, G569S, and [I66M + I289V], NOEV treatment of GALC‐transfected cells resulted in an increase in GALC processing, suggesting that these mutant forms were successfully trafficked to the lysosome. Unfortunately, the observed increase in cleaved product was not detectable in patient cells, and only one patient sample showed a statistically significant increase in enzyme activity following NOEV treatment. As expected from its chemical structure and ability to inhibit GALC, docking model analysis predicted that NOEV would bind the GALC active site. However, it is unclear how its affinity for GALC compares with other azasugars because NOEV lacks a nitrogen atom in the equivalent position shown to be required for high‐affinity binding of the nonalkylated azasugars. However, although the lack of nitrogen in the ring may lower its affinity for GALC, this feature may be an advantage in terms of exploiting the acidic conditions in the lysosome for chaperone dissociation. Predictions of binding free energy of NOEV to β‐gal at pH 5 and pH 7 suggest that the affinity was lower at pH 5 (Suzuki et al., 2009). This may also be the case for GALC and may prove to be a greater advantage than high‐affinity binding alone. Although NOEV has been identified as a chaperone for GM_1_‐gangliosidosis, Hossain et al. (2015) did not directly compare the potency of NOEV as a chaperone for GALC with the lysosomal β‐gal responsible for GM_1_‐gangliosidosis and, as such, the specificity for use as a Krabbe therapeutic remains unclear.

### Allosteric Chaperones

To date, the search for new candidate PCT molecules for KD has focused on the ability of small molecules to inhibit GALC. Although active‐site‐directed chaperones confer specificity, the counterintuitive use of enzyme inhibitors is often criticized because the chaperone is, by nature, a competitive inhibitor of its intended enzyme target. The requirement to balance between chaperoning activity and enzyme inhibition is a potential barrier to their therapeutic efficacy. To overcome problems associated with active‐site‐directed inhibitors, allosteric chaperones have been explored as an alternative chaperone strategy. By binding to an allosteric site, the functional state of the enzyme may be rescued without competitive inhibition.

Two molecules that were originally identified as weak inhibitors of GALC may confer some chaperoning activity via allosteric mechanisms rather than by active site binding. α‐Lobeline, a simple alkaloid, has been tested in two separate studies to identify candidate GALC chaperones (Fig. 3). First reported by W.C. Lee et al. (2010), α‐lobeline was identified as a weak GALC inhibitor by an enzyme activity screening method. Those molecules that inhibited GALC function by more than 25% were selected, and dose–response assays were used to confirm and validate the initial results. At the highest concentration of α‐lobeline tested (240 μM), both intra‐ and extracellular activity of the Krabbe‐associated hyperglycosylation mutation D528N increased in a neuronal cell line. The high dose of α‐lobeline required to cause an observed chaperoning effect is concerning because it has been suggested that application of more than 20 μM of compound will not easily pass the ER barrier (Butters et al., 2005). Furthermore, compounds at such high doses may elicit unwanted side effects elsewhere and are therefore not attractive leads for pharmaceutical development. Although other Krabbe‐associated mutations were tested (I234T and L629R), only D528N showed any rescue of function. However, given the number of missense mutations that exist in KD, those tested constitute only a small fraction of causative mutations and may not be true representations of the percentage of α‐lobeline‐responsive GALC candidates. Despite the use of image‐based immunostaining to localize GALC and characterize the mutants used in this study, immunolabeling and lysosomal GALC processing were not used as readouts of compound efficacy but might have proved useful for better understanding the effect of α‐lobeline on the cellular localization of mutant GALC.

Just as in W.C. Lee et al. (2010), Berardi et al. (2014) used an enzymatic assay to screen a series of molecules, including azasugar analogs, for inhibitory effects on GALC. Their initial screen did not identify any compounds with significant inhibition of GALC activity at the test concentration of 400 μM; despite this, studies were continued with two test compounds, α‐lobeline (identified previously) and 3′4′7‐trihydroxyisoflavone (Fig. 3). To test the effectiveness of these candidate chaperones, fibroblast cell lines carrying a range of clinically relevant missense GALC mutations (G537R, [E114K + N279T], and G41S) were treated with either α‐lobeline or 3′4′7‐trihydroxyisoflavone for 72 hr at 50, 100, and 200 μM before GALC activity was measured from whole‐cell lysates. In the cell line homozygous for the G537R mutation, α‐lobeline increased GALC activity at 50 and 100 μM, with no further increase in activity at 200 μM, whereas increasing concentrations of 3′4′7‐trihydroxyisoflavone increased GALC activity. Both α‐lobeline and 3′4′7‐trihydroxyisoflavone increased GALC activity in the cell line expressing both E114K and N279T mutations, with the greatest effect observed at lower concentrations of both candidate chaperones. Similarly, both α‐lobeline and 3′4′7‐trihydroxyisoflavone increased GALC activity in the cell line homozygous for the G41S mutation. For all these assays, GALC activity was compared with untreated cells but was not compared with normal WT GALC activity levels. Because each mutant cell line possessed different levels of residual GALC activity, it remains unclear which treatments brought activity up to a significant proportion of WT levels. Two late‐onset mutations not predicted to affect the fold of GALC were additionally analyzed, and no change in GALC activity was observed with either of the test compounds. This underscores the requirement of identifying the specific GALC variants that result in protein misfolding for successful testing and development of PCTs. This, alongside the difference in compound efficacy on those mutations tested, signifies the requirement for a tailored and individualized approach to chaperone therapy.

Both compounds explored showed weak GALC inhibition at pH 5.2, and docking analysis identified nine different potential binding sites on GALC, with only one site overlapping with that of the natural substrate. Thus, although these molecules were selected based on their capacity to inhibit GALC, the likely mechanism by which these molecules may function as PCs is via allosteric binding. One of the two molecules tested in this study, 3′4′7‐trihydroxyisoflavone, has been shown to possess inhibitory activity against other cellular enzymes, including cyclin‐dependent kinases, phosphatidylinositol 3‐kinase, xanthine oxidase, and macrophage migration inhibitory factor (Orita et al., 2001; Park et al., 2008; D.E. Lee et al., 2010). This, combined with the relatively low binding energies reported from the docking studies, raises some concerns with respect to the specificity of this molecule as a GALC chaperone.

Cell‐based high‐throughput screens with chemical libraries have also been undertaken to identify small‐molecule chaperones suitable for KD. Ribbens et al. (2013) successfully automated and optimized a highly sensitive fluorimetric assay for the identification of GALC inhibitors in a cell line expressing GALC mutation G270D, associated with late‐onset forms of KD. Unfortunately, none of the 1,280 molecules tested in this study showed any statistical improvement in GALC activity, and toxic effects confounded many of those that were tested. The extensive toxicity observed highlights a caveat of testing chaperones that are not directed against the active site because they can be confounded by off‐target and often detrimental effects. Moreover, many of the compounds included in the screens had not previously been indicated to cross the BBB. In light of these observations, for initial screening purposes, if not performing active‐site‐targeted drug design, it would be reasonable to repurpose approved drugs with a known ability to cross the BBB rather than screening large libraries of drugs that may never be relevant for CNS diseases. The use of preexisting drugs (such as NOEV and α‐lobeline) as candidate GALC chaperones is advantageous because many of their characteristics, such as low toxicity and the ability to penetrate the BBB, are already well defined and are critical characteristics when targeting neurological symptoms prominent in KD.

Although this study provides a useful developmental approach for applying high‐throughput methodologies, currently it allows only for single mutation testing. It does, however, allow for the possibility of identifying more general proteostasis regulators that promote enhancement of residual GALC–G270D activity via diverse mechanisms that may be missed in conventional rational drug design strategies.

## METHODS FOR MONITORING THE EFFECTIVENESS OF CHAPERONES

Just as with other LSDs, the effectiveness of chaperone molecules is commonly measured only by their capacity to increase the level of enzyme activity above a predefined threshold in treated cells. In the case of KD, enzyme activity does not correlate well with the severity of disease, meaning that conclusions drawn on activity alone are not as compelling as those studies that can illustrate increased stabilization of GALC, detection of cleaved GALC (indicating successful delivery to the lysosome), improved lysosomal localization, or reduced accumulation of psychosine (Wenger et al., 2000; Duffner, 2009). However, these alternative measures are considerably more difficult to carry out and are not easily amenable to high‐throughput approaches.

As outlined by Valenzano et al. (2011), it is essential to meet a number of key criteria to assess correctly the efficacy of a chaperone on its intended enzyme target. First, it is imperative to show that the observed effects are directly facilitated by the chaperone, commonly shown by stabilization or inhibition of either WT or mutant enzyme. Second, it is important to measure the effect of the chaperone on the level of total enzyme within the cell; for example, protein levels can be directly monitored with Western blotting. Finally, from the principle of chaperone therapy, candidate molecules should not only bind and stabilize GALC but also correct aberrant localization. To measure the ability of a chaperone to promote lysosomal trafficking, cell‐based methods such as subcellular fractionation and image‐based subcellular location can be carried out. This latter approach has the benefit of simultaneously monitoring the target enzyme and organelle‐specific markers as well as the potential to be adaptable for high‐throughput screening methodologies (Starkuviene and Pepperkok, 2007; Zanella et al., 2010).

For those missense mutations that retain residual activity but are mislocalized, chaperones may facilitate the transport of functional enzyme to its desired site of action within the lysosome, without directly increasing its activity. Therefore, although it is important to know whether a mutation retains activity for the success of a PCT candidate to be explored, it is also essential to understand how specific GALC mutations affect localization and functionality within the cell.

Although enzyme activity has limitations as a measure of chaperone efficacy, it is a commonly used approach that provides a relatively clear functional readout that is straightforward to perform. Because of its wide application, it is important to discuss a number of caveats to this approach that must be considered when analyzing activity data.

### Substrate Selection

In the work published to date, a range of different substrates has been used to monitor GALC activity, including tritiated GalCer (Biela‐Banas et al., 2014), alkylated fluorogenic and colorimetric substrates such as 2‐hexadecanoylamino‐4‐nitrophenyl‐β‐D‐galactopyranoside (HNG; W.C. Lee et al., 2010) and 6‐hexadecanoylamino‐4‐methylumbelliferyl‐β‐D‐galactopyranoside (HMG; Ribbens et al., 2013; Berardi et al., 2014; Hossain et al., 2015), and water‐soluble fluorogenic and colorimetric substrates such as 4NBDG (Hill et al., 2015) and 4MBDG (Martino et al., 2009).

Unfortunately, the activities determined with different substrates cannot be directly compared, making useful comparisons among studies impossible. Furthermore, the specificity of these substrates may differ and can be highly dependent on the assay conditions. For example, the concentration of taurocholate and oleic acid in the enzyme activity buffer is critical for the alkylated substrate HMG to establish specificity for GALC vs. β‐gal. Importantly, at high concentrations of taurocholate, HMG shows no specificity for GALC highlighted by the observation of equivalent enzyme activity in control and twitcher mouse samples (Wiederschain et al., 1992).

### Enzyme Source

The source of enzyme for most activity assays is whole‐cell lysate. Because many of these assays are often performed without any purification or enrichment steps, considerable activity is likely to be contributed by other galactosidase enzymes. Although this can be controlled, if the proportion of activity contributed by GALC is small and, thus, there is a large background correction the effectiveness of chaperone molecules may be under‐ or overestimated. Related to this, GALC has a very specific pH‐dependent activity profile, so it is critical that the activity be measured within the optimal pH range of the enzyme (pH 4.3–5.3; Hill et al., 2013). Outside of this pH range, GALC activity is significantly reduced such that enzyme activity measurements from whole‐cell lysates at suboptimal pH possess essentially no activity from GALC. An additional consideration for assays conducted on whole‐cell lysates is the difficulty of distinguishing among protein localized in different subcellular compartments, such as in the ER or endocytic/lysosomal compartments. The goal of PCT is to ensure that active enzyme is delivered to the required site of action in the lysosome, and measures of whole‐cell activity cannot verify the localization of this activity. In support of this, activities measured from samples enriched for the lysosomal fraction have shown a better correlation with disease severity than had been previously observed (Shin et al., 2016). This finding provides a good foundation for improved reliability for future activity measurements. Together with Wenger et al. (2014), who showed that in vitro activity assays might not give a true representation of the enzyme activity in relevant tissues such as the nervous system, these indicate that, although activity assays from whole‐cell lysates provide a reasonable first approximation of the effectiveness of any therapeutic, they do not provide information on either the cellular location or the functionality of the chaperone. Therefore, it is important that alternative measures of efficacy, such as restored GALC processing, lysosomal localization, or reduced psychosine accumulation, be carried out for a more robust identification of effective chaperone molecules.

### GALC Quantification

To distinguish between absence of protein and inactive protein, it is important to be able to estimate the amount of GALC present in an activity assay. This is especially important when comparing relative activities among different mutations or between PCT‐treated and ‐untreated cells. Accurate quantification of very small amounts of GALC is very challenging, not just because of detection limits in techniques such as Western blotting but also because of the presence of both full‐length and cleaved forms of GALC within cells. Moreover, a critical consideration for any activity assay performed on drug‐treated cells is the addition of a washout prior to activity measurement to prevent the carryover of any residual PCs. However, the length of washout required is dependent on the half‐life of the enzyme, and, in many cases, this differs between WT and mutant enzymes.

## IDENTIFYING CANDIDATE MUTATIONS FOR PCT

The development of successful PCT for KD relies not only on the robust identification of candidate chaperones but also on the identification of those mutations that will respond best to treatment. PCT is not a one‐size‐fits‐all therapy and requires an understanding of the mechanism of the defect caused by specific mutations to allow the best chance for successful treatment. More than 110 mutations have been identified in the GALC gene that affect GALC mRNA processing or cause deletions, frame shifts, and missense mutations (Rafi et al., 1996; Wenger et al., 1997; De Gasperi et al., 1999; Lee et al., 2006; Tappino et al., 2010). It is evident that, for those cases of KD resulting from the common 30‐kb deletion within the *GALC* gene or arising from mutations in cofactors such as saposin A, PCT will not be an appropriate therapy (Luzi et al., 1995; Rafi et al., 1995; Spiegel et al., 2005). Instead, PCT should be targeted at those mutations that result in defective cellular localization resulting from compromised enzyme stability or folding. The nature and location of the amino acid substitution will play a critical role in determining whether a variant is likely to affect folding and, therefore, respond to PCT.

**Figure 4:**
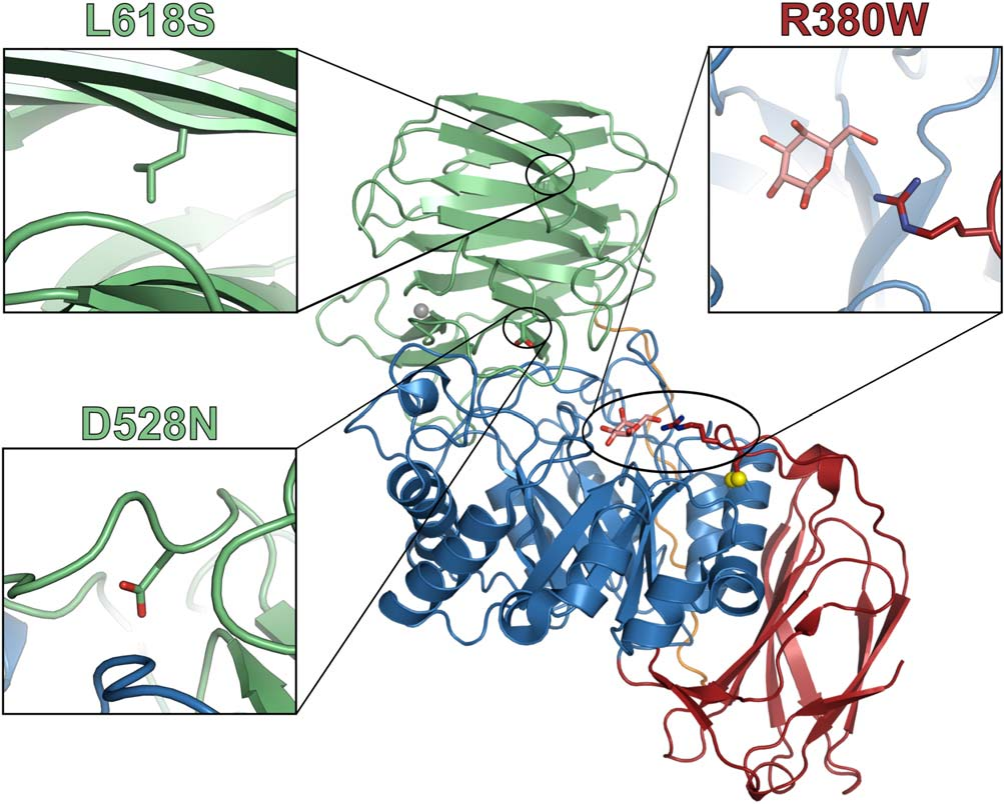
KD‐associated mutations of GALC. Three residues that are mutated in KD are highlighted on the structure of GALC (PDB ID: 3ZR6). The structure is colored according to domain (TIM barrel, blue; β‐sandwich, red; lectin domain, green), and the disulfide bond (yellow) and calcium ion (gray) are illustrated as spheres. The galactose product (pink sticks) is shown in the GALC active site. For each mutation, the closeup view (**inset**) shows the relevant residue as sticks (oxygen atoms, red; nitrogen atoms, blue) and the surrounding region of the structure that would be affected by the mutation.

Within the subset of missense mutations, aberrant GALC function may result from catalytic inactivity, posttranslational modifications, misfolding, or premature degradation. Structural data can provide a framework for predicting the mechanisms of certain mutations to help identify those mutations that may be responsive to PCT. Missense mutations that lie within the active site of GALC, such as the R380W mutation (Fig. 4), directly disrupt the interactions between enzyme and substrate, critically affecting enzyme activity but not folding (Hill et al., 2013; Spratley, et al., 2016). The R380W mutation thus causes a severe, early‐onset form of KD because of catalytic inactivity (Wenger et al., 1997). Enzyme stabilization by a PC cannot overcome this type of defect, so mutations of critical active site residues will not respond to PCT. It is important to recognize that this mechanism of loss of GALC activity is distinct from those caused by folding defects.

It is likely that some chaperones will exhibit stabilizing effects in a highly mutation‐specific manner. This may be the case with the GALC mutation D528N, which showed enhanced activity when treated with α‐lobeline (W.C. Lee et al., 2010). The mutation of D528 to asparagine (N) altered the posttranslational modification of GALC by introducing an additional N‐linked glycan. Because D528 lies on a surface loop within the lectin domain, the introduction of an extra glycan may not have caused significant misfolding (Fig. 4). Thus, for this mutation, the chaperoning effect conferred by α‐lobeline might have been specific to the newly introduced glycan. In support of this, it was observed that α‐lobeline does not confer any increase in thermal stability to WT GALC and may also explain why it did not have a chaperoning effect on the other GALC variants tested (W.C. Lee et al., 2010; Hill et al., 2015).

Identifying the best candidates for PCT is challenging, even with predictions made possible with reference to the GALC atomic structure. A large proportion (∼80%) of the identified KD missense mutations are buried deep within the structure and are predicted to cause GALC misfolding (Deane et al., 2011). Because all three domains of GALC contribute to the formation of the active site pocket, it is possible that even localized misfolding of any region could perturb enzyme activity resulting from disruption of the active site architecture (Deane et al., 2011). Therefore, those mutations that have been identified as catalytically inactive but are not themselves localized close to the active site may benefit from overall enzyme stabilization conferred by a chaperone and should not be discounted as targets for PCT. One example of a GALC mutation that is buried in the structure and lies far from the active site is L618S (Fig. 4). This mutation is found within the lectin domain, has been shown to have very low activity, and is implicated in late‐infantile‐ and adult‐onset KD (Furuya et al., 1997; Satoh et al., 1997; Xu et al., 2006). Although mutation of L618 to serine (S) does not introduce a significantly larger side chain that might cause steric clashes, it alters the hydrophobic nature of this side chain and, thus, alters the local interactions in the lectin domain. The mutations responsible for later‐onset KD, such as L618S and G270D, may possess subtler misfolding defects than the early‐onset variants and, therefore, may respond better to PCT. Therefore, these might turn out to be important variants to consider in high‐throughput screening of new chaperone candidates. Correct identification of misfolding mutations in KD patients will identify suitable KD candidates for PCT. However, it is likely that only a fraction of missense mutations responsible for GALC misfolding may be amenable to PCT, exemplified by the mutation‐specific effects observed for PC candidates tested to date.

An important consideration when characterizing mutations is the wide range of GALC activity reported in noncarrier and carrier individuals (Harzer et al., 2002). This has, in part, been attributed to polymorphisms present in the normal allele of the GALC gene that contribute to lower‐than‐normal GALC activity and can confound diagnosis and analysis of therapeutic efficacy (Furuya et al., 1997; Wenger et al., 2014; Shin et al., 2016). The G270D mutation, commonly associated with late‐onset forms of the disease, is often found alongside the common polymorphism I546T (found in 35–45% of the population); together, they show lower GALC activity than when expressed on their own (Wenger et al., 2014). Likewise, it has been shown by Shin et al. (2016) and Spratley et al. (2016) that the combination of certain cispolymorphisms and disease mutations can significantly reduce the activity and trafficking of GALC. The full impact of known polymorphisms on KD prognosis and therapeutic intervention has yet to be elucidated but must be taken into consideration when characterizing and identifying candidate GALC mutations. Alongside the polymorphic background of each Krabbe variant, heterogeneity makes for complex analysis of disease phenotype and can also confound interpretation of chaperone effects. For heterozygous KD mutations such as [E114K + N279T], determining the chaperone‐responsive mutation is difficult, and it is currently unclear whether the presence of another mutation alters a chaperoning effect (Grabowski, 1997).

### Validating Candidate Mutations

After a suitable mutation has been identified as a target for PCT, ongoing validation in an appropriate model system should be pursued. However, chaperones that appear mutation specific are currently not easily explored in living models of the disease. Confounding this is the small Krabbe patient population, which means that patient tissue samples are difficult to obtain, especially in the early stages of novel chaperone development. Most preliminary testing is, therefore, conducted in cell lines expressing clinically relevant mutations. Although this has been shown to be effective for initial screening, it may not translate directly to the clinical phenotype.

Several spontaneous models for KD exist in a number of species, including mouse (Suzuki and Suzuki, 1995), dog (Victoria et al., 1996), and rhesus monkey (Baskin et al., 1998). Among these, the twitcher mouse is the most commonly studied. However, as a mimic of the 30‐kb deletion, the lack of GALC is inappropriate for analysis of PCT efficacy for missense mutations (Kobayashi et al., 1980; Suzuki and Suzuki, 1995). The *GALC* ^*twi‐5j*^ mouse has a spontaneous missense mutation in GALC equivalent to the human GALC substitution E130K responsible for a severe infantile form of the disease and represents a more appropriate model for drug screening (Tappino et al., 2010; Potter et al., 2013). Although spontaneous model systems provide tools for investigating pathogenesis and evaluating therapeutic approaches, the ability to introduce specific clinical mutations into appropriate model systems quickly and effectively will allow more effective evaluation of potential therapeutics and validation of PCT candidates for KD. This has been made feasible by advances in gene editing techniques such as CRISPR‐cas, zinc‐finger nucleases, and transcription‐activator‐like effector nucleases, all of which provide strategies for achieving site‐specific gene manipulations to accelerate the generation of new mutation‐specific models (Cui et al., 2011; Yang et al., 2014; Sommer et al., 2015). Although these techniques have been shown to be effective in generating mutation‐specific mouse models and to have the potential to extend into larger animal models to study the neurodegenerative aspects observed in KD, the high associated cost and time to generate such models are still limiting (Inui et al., 2014; Tu et al., 2015). To overcome this, studies of GALC in zebrafish (*Danio rerio*) provide an affordable alternative platform in which to evaluate therapeutic approaches effectively (Zizioli et al., 2014). Generation of site‐specific mutations can be carried out efficiently with the gene‐editing techniques mentioned above and are well established in this model system (Huang et al., 2012; Hwang et al., 2013).

## COMBINATION THEREAPY: ENHANCING FUTURE THERAPIES

PCs have shown great potential when used in combination with other therapeutic approaches. In LSDs for which enzyme replacement therapies are available, synergistic effects have been observed when PC candidates are combined with recombinant enzymes (Parenti, 2009; Lukas et al., 2015). This combination approach is known as *enzyme enhancement therapy* and has the advantage that PCs are used with the exogenous WT enzyme, and, consequently, the effects observed are independent of the mutation carried by the patient and may translate to improved numbers of responsive patients. Although the use of ERT in CNS‐involved LSDs such as KD is limited to the management of associated peripheral dysfunction (Platt, 2014), it was shown in the twitcher mouse model of KD that both peripheral and cerebral administration of GALC significantly increased life span, improved motor function, and reduced psychosine accumulation (Lee et al., 2005; Qin et al., 2012). Although ERT has not yet been clinically developed for KD, a major therapeutic goal is the development of strategies for improving delivery of peripherally infused enzyme to the brain and the CNS. Several approaches are currently being developed in related diseases to facilitate the transport of enzyme across the BBB (Zhang and Pardridge, 2005; Grubb et al., 2008; Osborn et al., 2008; Gabathuler, 2010). Future development of ERT for KD will benefit from the identification of stabilizing PC molecules.

PC molecules may enhance ERT by several mechanisms, including increasing the half‐life and the biodistribution of administered enzyme. Poor biodistribution is a significant obstacle for ERT, and, even if the enzyme reaches the appropriate tissue, it has been suggested that recombinant enzymes may be relatively unstable when exposed to suboptimal environments on transit to the lysosome (Schoser et al., 2008; Benjamin et al., 2012). Coadministration of recombinant enzyme and active‐site‐directed chaperones N‐butyl deoxynojirimycin (NB‐DNJ) and isofagomine used in Pompe's and Gaucher's disease, respectively, showed improvement in stability, lysosomal trafficking, maturation, and intracellular activity (Shen et al., 2008; Porto et al., 2009). Similar synergistic effects on α‐glucosidase (GAA), the defective enzyme in Pompe disease, were observed with the allosteric chaperone N‐acetlycysteine (NAC; Porto et al., 2012). ERT is often used in Pompe's disease but has shown limited therapeutic efficacy in some patients (Porto et al., 2009). NAC improved the stability of GAA as a function of pH and temperature, with no effect on activity. As an allosteric binder, NAC does not interact with the catalytic domain and is therefore not a competitive inhibitor of the enzyme. Furthermore, coadministration of GAA and NAC improved GAA activity in a dose‐dependent manner and additionally improved correction of GAA deficiency in patient cell lines with mutated GAA. Although Pompe's disease is primarily a systemic disease, these proof‐of‐principle experiments indicate the potential use for chaperones as stabilizing agents for administered recombinant enzymes used in ERT. The combination of NAC and DNJ (an active‐site‐directed chaperone) gave the highest thermal stability of GAA. Because these two small molecules have different chaperone profiles, this may overcome some of the mutation‐specific effects and the complexity observed in compound heterozygous patients.

Moreover, it has been observed that enzymes used in ERT can destabilize while in storage, and, in response to the administered unfolded protein, a hypersensitive immune reaction can be triggered (Maas et al., 2007). Therefore, inclusion of a chaperone may limit protein unfolding by stabilizing the ERT enzyme in storage, increasing efficacy and limiting adverse effects observed in ERT. Thus, in addition to their role in rescuing destabilized mutant enzymes that are retained in the ER, the small molecules reviewed in this work may enhance the effectiveness of recombinant WT enzymes in future ERT development programs for KD.

Currently, the only available treatment for KD is HSCT, which slows the progression of disease when performed before the onset of symptoms (Escolar et al., 2005). For the infantile form of KD, initiating treatment sufficiently early is highly challenging. It is possible that small molecules (such as those reviewed here) could be administered before the onset of KD symptoms to help lengthen the therapeutic window for HSCT and enhance the rate of success.

### A Bifunctional Role for Chaperone Molecules

In the case of PCT molecules that mimic the substrate, such as azasugar derivatives, it is conceivable that their effects are bifunctional. The similarity of these PCT molecules to the glycan on the substrate means that, in addition to their ability to bind their respective hydrolases, they also have the potential to bind the glycan‐synthesizing enzyme. For example, miglustat (also known as *zaveska*) is NB‐DNJ, an alkylated glucose analog of DGJ, and is used for the treatment of adult patients with mild to moderate type I (nonneuropathic) Gaucher's disease. Miglustat is thought to function primarily as an SRT by inhibiting the glucosylceramide synthase (Cox et al., 2000, 2003; Moyses, 2003). However, because this molecule also binds to the active site of acid β‐glucosidase and stabilizes the enzyme, it may also function as a PCT (Brumshtein et al., 2007; Abian et al., 2011). Similarly, migalastat is the therapeutic name for DGJ and has shown effectiveness as a PCT for Fabry's disease by stimulating the activity of α‐galactosidase and decreasing accumulation of substrate in female patients (Giugliani et al., 2013). Alkylated derivatives of DGJ have been identified as inhibitors of glycolipid biogenesis, so it is possible that some PCT molecules also function as SRTs by inhibiting the relevant synthetic enzyme (Platt et al., 1994). This potential for PCT molecules to function in other pathways that ameliorate disease may also be relevant for some molecules that do not function by binding the active site. For example, isoflavones similar to 3′4′7‐trihydroxyisoflavone described by Berardi et al. (2014) have been identified as SRT agents in mucopolysaccharidoses (Piotrowska et al., 2006; Arfi et al., 2010; Kloska et al., 2011).

### Targeting Proteostasis

The combination of PCs with other molecules that promote general protein folding has shown synergistic effects in Gaucher's‐ and Tay Sachs‐derived patient fibroblasts (Mu et al., 2008b). The proteostasis regulator celastrol was used in combination with the PC NN‐DNJ and enhanced the restoration of mutant enzyme function. Proteostasis modulators that affect calcium flux, such as the L‐type calcium channel blockers diltiazem and verapamil, have been shown to enhance enzyme function in fibroblasts from patients with Gaucher's disease, α‐mannosidosis, and type IIIa MPS (Mu et al., 2008a; Ong et al., 2010). Diltiazem inhibits and stabilizes glucocerebrosidase at ER pH; therefore, as well as acting as a general proteostasis modulator, this may be acting directly as a PC in this setting (Rigat and Mahuran, 2009). This class of therapeutic increases the ability of the cell to cope with misfolding‐prone proteins and may prove useful in targeting a range of different mutations or diseases, helping to overcome many of the mutation‐dependent chaperone effects currently observed.

## FUTURE CHALLENGES

Despite the recent progress in identifying and characterizing small molecules that elicit chaperoning effects on GALC in vitro, significant challenges still exist that must be overcome to translate PCs into viable treatment options for those who suffer from KD. The significant CNS involvement poses one of the greatest challenges in the development of KD therapeutics. PCT is one strategy that has the potential to overcome this; however, the limitations in our current understanding of both the mechanisms of chaperone–enzyme interaction and the underlying causative mechanisms associated with specific mutations in KD sufferers highlight the requirement for additional investigation. Specific concerns relating to those chaperones investigated to date for KD are listed below.

### GALC Specificity

Although specificity for beta‐configured galactosidases has been shown for active‐site‐directed chaperones, obtaining specificity for GALC vs. β‐gal has proved to be extremely difficult. Evaluating this specificity relies on the use of both appropriate assays and substrates when measuring the chaperone response. Also, because of the structural similarity of the active sites of these two enzymes, to confer this specificity it may be essential to focus on modifications of the aglycone region of PC candidates rather than the azasugar portion.

### Off‐Target Effects of Allosteric Chaperones

To overcome problems experienced with inhibition of the target enzyme, nonactive site inhibitors are used; however, these have the potential to elicit significant off‐target effects. Because these are directed away from the active site, off‐target effects are difficult to predict, monitor, and overcome. During chaperone development, the dosing strategy may become particularly important when using this class of chaperone.

### Mutation‐Dependent Chaperone Effects

It is likely that only a subset of missense mutations will respond to specific chaperones. There are two major implications of this. First, candidate chaperones may be missed if the number of mutations being screened is not sufficiently large or if the selection of potentially responsive mutations is not appropriately made. Second, development and administration of multiple therapeutic interventions may be required for successful treatment. As an alternative strategy, proteostasis regulators, used carefully, may represent a broad‐spectrum therapy that could overcome mutation‐dependent effects.

### Measurement of Chaperone Efficacy

Currently, increased activity is the main readout of chaperone efficacy, which may be misleading. A clear readout of improved lysosomal delivery of functional material is required to assess comprehensively the effectiveness of PCT. Measuring the effectiveness of any of the chaperones discussed here requires the development of alternative assays that are more amenable to high‐throughput approaches than are currently available.

## CONCLUSIONS

The past decade has seen PCT emerge as a viable and promising therapeutic option for LSDs. The dominating presence of CNS pathology in KD has undoubtedly hindered the acceleration of research and clinical translation in this area with respect to other non‐CNS LSDs. Despite this, the use of PCT for KD does show promise, and several GALC candidate chaperones have been identified. Because of the difficulty of establishing a clear genotype–phenotype correlation, it is clear that a detailed mechanistic understanding of both the candidate chaperone and the underlying target mutation will allow enhanced translation of successful therapy. However, it is evident that the measurement of chaperone efficacy should be based on several complementary biological assays that monitor the ability of a chaperone not only to increase enzyme activity but also to promote correct trafficking to the lysosomal compartments. Therefore, although increased GALC activity is an important readout, we believe it should not be the sole determinant of a successful chaperone. The parallel development of multiple therapeutic approaches is likely to be the most successful way to target the complex phenotypes observed in KD, and the identification of new PCT candidates is a critical contribution to future therapeutic interventions for this debilitating disorder.

## CONFLICT OF INTEREST STATEMENT

The authors declare that they have no conflicts of interest associated with the content of this Review.

## ROLE OF AUTHORS

Both authors had full access to the data presented in this Review and take responsibility for the integrity of the data and the accuracy of the data interpretation. Both authors contributed to the study concept and design and to the drafting and critical review of the Review.
